# Dueling Malignancies: A Rare Case of Concurrent Chronic Lymphocytic Leukemia (CLL) and Chronic Myeloid Leukemia (CML) in a Multimorbid Elderly Patient

**DOI:** 10.7759/cureus.105422

**Published:** 2026-03-18

**Authors:** Meshal Alzahim, Ahmed Alakedi, Omar Almugren, Osama Khoja, Nahla Alias, Alfatih Abdelgader

**Affiliations:** 1 Pathology, King Saud University, Riyadh, SAU; 2 Laboratory Medicine, Dr. Sulaiman Al Habib Medical Group, Riyadh, SAU

**Keywords:** chronic lymphocytic leukemia (cll), chronic myeloid leukemia (cml), heme-onc, multi pathologies, rare cancers

## Abstract

The concurrent presence of multiple hematological malignancies in a single patient is exceptionally rare. Coexisting chronic lymphocytic leukemia (CLL) and chronic myeloid leukemia (CML) in an elderly patient with multiple comorbidities presented unique diagnostic and management challenges.

We report a 69-year-old female with a history of type 2 diabetes mellitus, hypertension, chronic kidney disease, and ischemic heart disease. The patient initially presented to an external hospital on 5 January 2025 with shortness of breath and decreased level of consciousness. Initial laboratory investigations at the referring hospital revealed critical values with hemoglobin of 2.9 g/dL and platelet count of 9 ×10⁹/L, requiring urgent transfusion support. During hospitalization at the referring hospital, she developed acute respiratory distress necessitating endotracheal intubation. She was subsequently referred to our institution on 9 January 2025 for further hematologic evaluation. Bone marrow examination showed hypercellularity (70-80%) with trilineage hematopoiesis. Flow cytometric analysis confirmed chronic lymphocytic leukemia (CLL). Cytogenetic studies demonstrated the Philadelphia chromosome in all analyzed metaphases, establishing the diagnosis of chronic myeloid leukemia (CML).

This case illustrates the rare simultaneous occurrence of CLL and CML in a patient with significant comorbidities and life-threatening cytopenia at presentation despite a history of leukocytosis and thrombocytosis. The case underscores the importance of comprehensive hematological evaluation, including flow cytometry and cytogenetic analysis, in patients with complex presentations. Managing patients with dual hematological malignancies requires careful consideration of treatment interactions, comorbidities, and complications, highlighting the need for a multidisciplinary approach to optimize outcomes.

## Introduction

Chronic lymphocytic leukemia (CLL) and chronic myeloid leukemia (CML) are distinct hematologic malignancies characterized by different cellular origins, molecular abnormalities, and clinical courses [1-3]. CLL is a lymphoproliferative disorder characterized by the accumulation of mature monoclonal B lymphocytes in the peripheral blood, bone marrow, and lymphoid tissues [1]. In contrast, CML is a myeloproliferative neoplasm driven by the BCR-ABL1 fusion gene, which results from the reciprocal translocation t(9;22)(q34;q11), known as the Philadelphia chromosome [2].

CLL represents the most common leukemia in adults in Western populations, with an estimated incidence of approximately 4-5 cases per 100,000 individuals annually [1]. CML accounts for approximately 1-2 cases per 100,000 individuals per year and constitutes approximately 15% of adult leukemias [2]. Although these malignancies are relatively common individually, the coexistence of both diseases in the same patient is extremely rare [4].

The simultaneous or sequential occurrence of CLL and CML has only been reported in a limited number of cases in the literature [4-6]. In most reported cases, the diseases develop sequentially, with CLL often preceding the diagnosis of CML, while concurrent presentation at the time of diagnosis is particularly uncommon [5].

Several mechanisms have been proposed to explain the coexistence of these two hematologic malignancies. These include the development of two independent malignant clones, transformation from a common pluripotent hematopoietic stem cell, or therapy-related clonal evolution following cytotoxic treatment [6]. Molecular studies in some reported cases suggest that the lymphoid and myeloid components may arise from distinct clonal populations, supporting the hypothesis of independent clonal origins [4].

Due to the rarity of this phenomenon, each additional reported case contributes valuable insights into the biological relationship between these two malignancies, as well as the diagnostic and therapeutic challenges they present. Here, we report a rare case of concurrent CLL and CML diagnosed in the same patient, highlighting the diagnostic findings and reviewing the relevant literature.

## Case presentation

Clinical history

We present the case of a 69-year-old female with a known history of type 2 diabetes mellitus, hypertension, chronic kidney disease (CKD), ischemic heart disease (IHD), and leukemia, who presented to an external hospital on January 5, 2025, with complaints of shortness of breath and decreased level of consciousness over the preceding days. Initial laboratory investigations revealed a hemoglobin level of 2.9 g/dL and platelet count of 9x10⁹/L, indicative of severe anemia and thrombocytopenia. During hospitalization, the patient received four units of packed red blood cells and six units of platelets. Subsequently, she developed acute respiratory distress; an echocardiogram was performed and found to be unremarkable. Hydrocortisone 60 mg IV OD was initiated, and the patient was intubated in preparation for transfer to a higher care facility. She was accepted for further management with a working diagnosis of severe anemia, thrombocytopenia, and leukemia.

Laboratory findings

General Lab Results

An initial complete blood count (CBC) performed at our institution revealed significant abnormalities consistent with hematologic malignancy. The patient presented with leukocytosis, evidenced by an elevated white blood cell count of 15.9x10⁹/L. Differential counts demonstrated a predominance of neutrophils 6.9x10⁹/L, lymphocytes 6.2x10⁹/L, and basophils 0.08 x10⁹/L, indicating potential leukemic activity. Red blood cell indices were also deranged, with anemia reflected by a low hemoglobin level of 6.5 g/dL. These findings are consistent with an evolving or active leukemic process, warranting further hematological evaluation and bone marrow biopsy for definitive diagnosis (Table 1).

**Table 1:** Patient laboratory results WBCs, white blood cells; RBCs, red blood cells; MCV, mean corpuscular volume; MCH, mean corpuscular hemoglobin; MCHC, mean corpuscular hemoglobin concentration; RDW, red cell distribution width; MPV, mean platelet volume; H, high; L, low

Test Parameter	Reference Range	January 9, 2025	February 4, 2025	April 22, 2025
Electrolytes & Metabolic Parameters				
Calcium (mmol/L)	2.15 - 2.55	1.97(L)	2.21	2.59
Magnesium (mmol/L)	0.7 - 1.05	1.12	0.95	0.85
Phosphorus (mmol/L)	0.74 - 1.52	1.42	0.97	1.04
Urea (mmol/L)	2.78 - 8.07	22.58 (H)	7.66	7.02
Creatinine (µmol/L)	44.2 - 106.1	142.1(H)	68.90	35.70
Sodium (mmol/L)	136 - 145	-	144	136
Potassium (mmol/L)	3.5 - 5.1	4.7	3.7	4.0
Chloride (mmol/L)	98 - 107	123(H)	109(H)	103
Bicarbonate (mmol/L)	23 - 31	23	24	24
Procalcitonin (ng/mL)	-	-	-	0.05 Negative
Complete Blood Count				
WBCs (×10⁹/L)	4 - 11	15.9	41.40(H)	18.30(H)
Neutrophils (×10⁹/L)	2 - 6.9	6.9	29.20(H)	10.20(H)
Neutrophils %	37 - 80	48.800	70.50	55.500
Lymphocytes (×10⁹/L)	0.6 - 4	6.2	8.170(H)	5.810(H)
Lymphocytes %	10 - 50	47.00	19.70	31.80
Monocytes (×10⁹/L)	0.2 - 0.9	0.250	1.450(H)	0.906(H)
Monocytes %	1 - 11	3.57	3.50	4.95
Eosinophils (×10⁹/L)	< 0.5	0.028	0.047	0.430
Eosinophils %	0 - 7	0.406	0.115	2.350
Basophils (×10⁹/L)	< 0.2	0.08	0.617(H)	0.255(H)
Basophils%	0 - 1	0.142	1.490(H)	1.390(H)
RBCs (×10¹²/L)	4.04 - 6.13	2.38(L)	3.55(L)	3.88(L)
Hemoglobin (g/dL)	11.8 - 14.8	6.5(L)	10.2(L)	10.7(L)
Hematocrit %	33 - 45	19.8(L)	32.9(L)	34.5
MCV (fL)	78 - 96	83.0	92.6	88.9
MCH (pg)	27 - 32	27.2	28.8	27.6
MCHC (g/dL)	29 - 37	32.7	31.1	31.0
RDW %	11.6 - 15.5	19.9(H)	17.5(H)	16.0(H)
Platelets (×10⁹/L)	150 - 450	14(L)	786(H)	482(H)
MPV (fL)	7.4 - 10.4	8.88	10.50(H)	10.20
C-reactive protein (mg/L)	< 5	123.8(H)	-	17.3(H)

**Figure 1:**
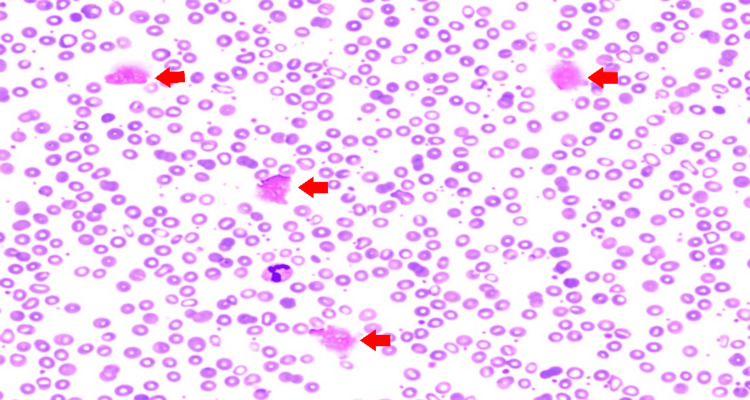
Peripheral blood smear demonstrating smudge cells (red arrows)

The peripheral smear showed moderate anisocytosis, ovalocytes, rare spherocytes, smudge cells, and left-shifted granulocytes, along with atypical lymphocytes and increased platelets with large forms (Figure 1).

Bone Marrow and Ancillary Studies

**Figure 2:**
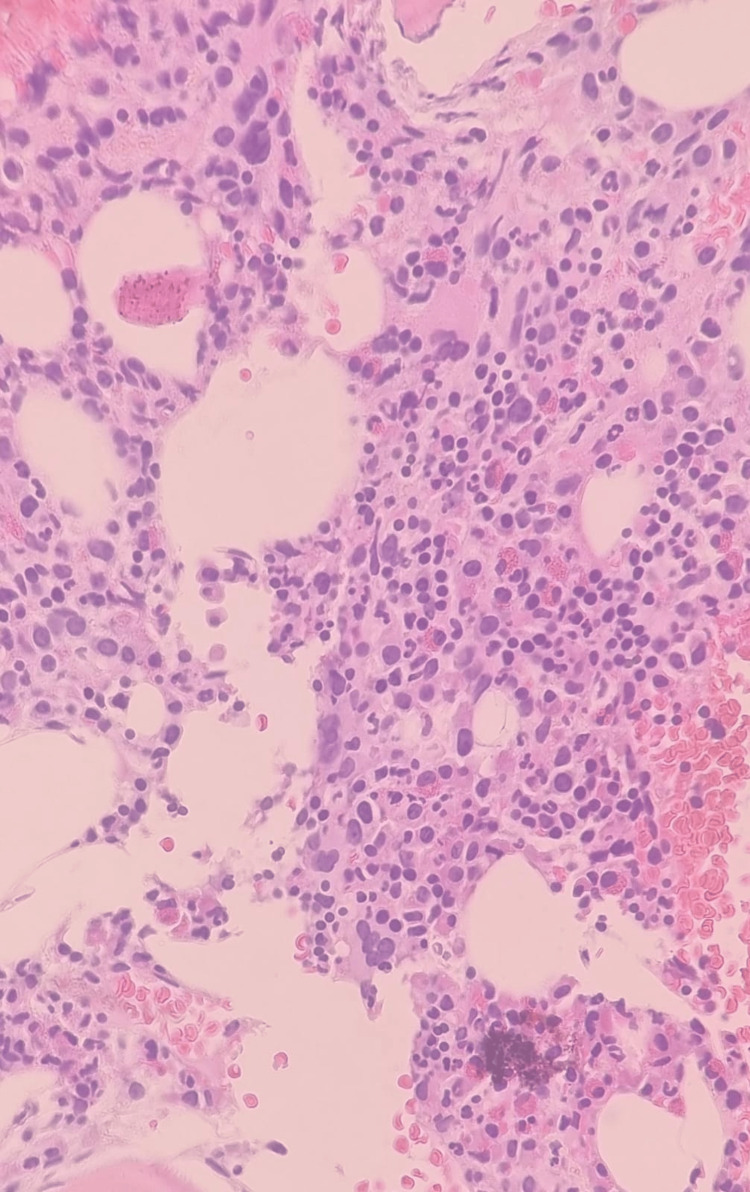
Hypercellular bone marrow biopsy (70-80% cellularity)

Bone marrow trephine biopsy from the right iliac crest revealed a hypercellular marrow with approximately 70-80% overall cellularity, which is considered elevated for the patient’s age (Figure 2). 

**Figure 3:**
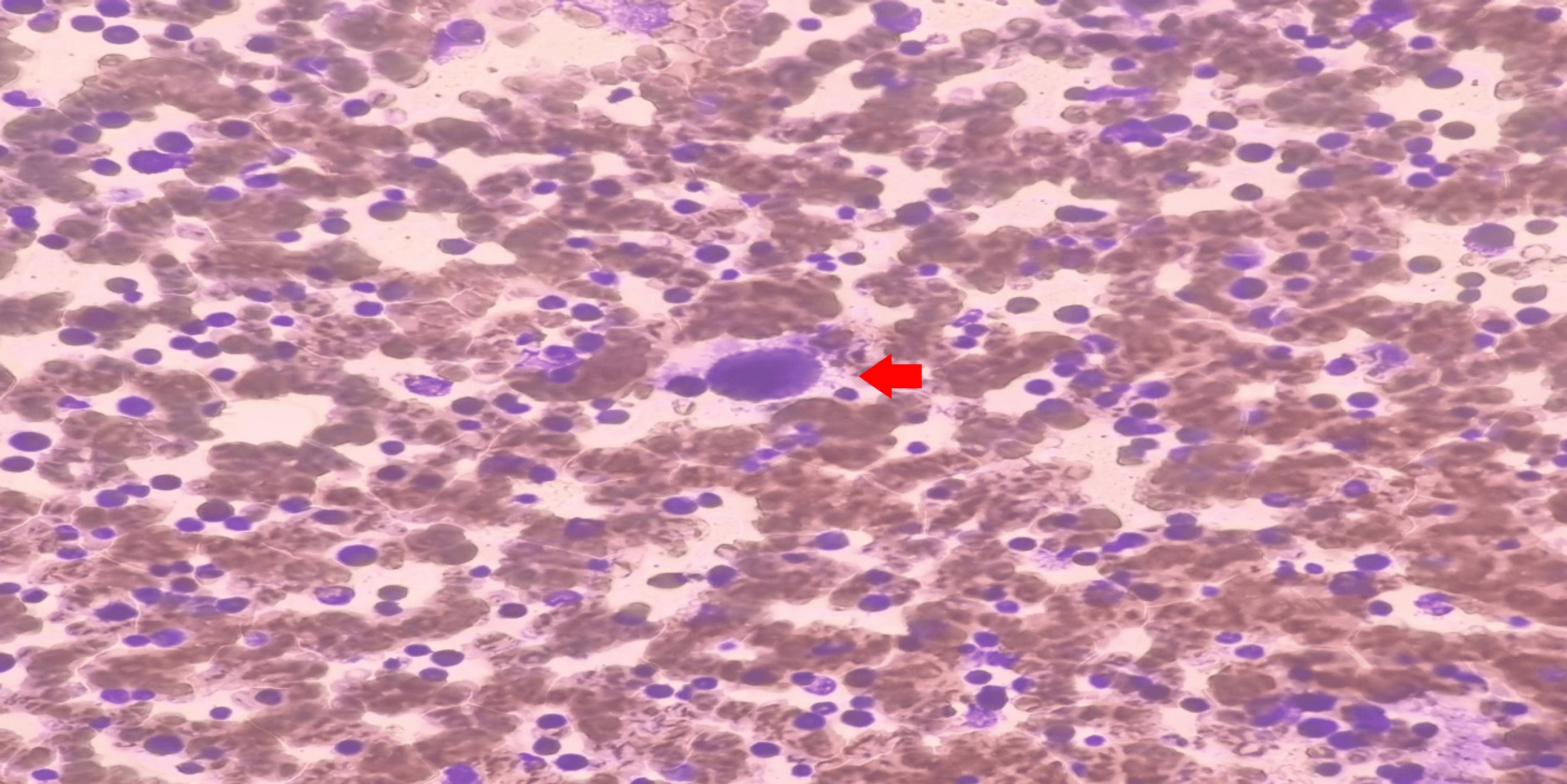
Bone marrow aspirate smear showing micromegakaryocyte (red arrow)

Both granulopoiesis and erythropoiesis were active, with adequate megakaryopoiesis and variable morphology (Figure 3).

The bone marrow aspirate demonstrated a normoblastic erythroid series and trilineage hematopoiesis with the following differential: 1% blasts, 4% promyelocytes, 12% myelocytes, 17% metamyelocytes, 28% segmented neutrophils and bands, 23% erythroid precursors, 13% lymphocytes, and 1% plasma cells.

Ancillary studies

Flowcytometry Findings

Flow cytometric analysis was conducted on bone marrow (BM) aspirate using a lymphoma-specific panel. Viability assessment showed 98.0% viable cells. The analysis identified an abnormal clonal B-cell population, characterized by positivity for CD5, CD19, CD23, CD25, CD43, CD79b, CD200, HLA-DR, and surface Kappa light chain restriction. CD20 and CD22 expression were dim. The population was negative for CD10, CD11c, CD34, CD38, CD103, CD117, CD123, and Lambda light chains. These immunophenotypic features are diagnostic of CLL and consistent with previous morphological and clinical findings. The absence of immature markers (e.g., CD34, CD117) and presence of mature B-cell markers support a mature lymphoid neoplasm (Table 2).

**Table 2:** Flow cytometry immunophenotype of abnormal lymphoid population CD, cluster of differentiation; FMC7, Fas membrane antigen 7; HLA-DR, human leukocyte antigen-DR isotype

Marker	Expression	Intensity
CD45	Positive	Bright
CD19	Positive	Moderate
CD20	Positive	Dim
CD5	Positive	Dim
CD23	Positive	Moderate
CD10	Negative	-
FMC7	Negative	-
CD3	Negative	-
CD34	Negative	-
HLA-DR	Positive	-
Kappa	Restricted	-
Lambda	Negative	-

Cytogenetic and Molecular Findings

Conventional karyotyping performed on bone marrow aspirate revealed an abnormal female karyotype, with a total of 20 metaphases analyzed. All metaphases demonstrated a balanced reciprocal translocation t(9;22)(q34;q11.2), consistent with the presence of the Philadelphia chromosome, confirming the diagnosis of CML. The diploid chromosome number was 46, XX. These findings corroborate the clinical suspicion of a myeloproliferative neoplasm and support the coexistence of CML in this patient, alongside chronic lymphocytic leukemia (CLL). A summary of the cytogenetic findings is shown in Table 3.

**Table 3:** Summary of cytogenetic findings ISCN, International System for Human Cytogenomic Nomenclature

Parameter	Result
Sample Type	Bone Marrow
Cytogenetic Abnormality	t(9;22)(q34;q11.2) – Philadelphia chromosome
Frequency of Abnormality	Detected in 100% of metaphases analyzed
Cytogenetic Interpretation	Abnormal female karyotype consistent with CML
ISCN Result	(46XX,t(9;22)(q34;q11.2)(20 metaphases

The molecular genetic (qPCR) revealed detectable amounts of BCR::ABL 1 fusion transcripts. 

Management and current treatment

The patient’s management plan is being guided by the dual diagnosis of CLL and CML, along with significant comorbidities, including type 2 diabetes mellitus, hypertension, ischemic heart disease, and chronic kidney disease. At the time of admission, the patient presented with symptomatic anemia and thrombocytopenia requiring urgent transfusion support. She received four units of packed red blood cells and six units of platelets. She later developed acute respiratory distress, necessitating intubation and intensive care management. At the time of admission, the patient presented with symptomatic anemia and thrombocytopenia requiring urgent transfusion support and received four units of packed red blood cells and six units of platelets. Her current medications include Exforge 10/160 mg orally once daily, chlorambucil 0.5 mg/kg orally once daily, hydroxyurea 500 mg orally twice daily, and imatinib 400 mg orally once daily.

## Discussion

The coexistence of CLL and CML in a single patient represents an uncommon clinical entity that raises important questions regarding the relationship between lymphoid and myeloid neoplasms. Although both disorders originate from hematopoietic cells, they arise from distinct cellular lineages and are driven by different molecular mechanisms [1,2].

CML is characterized by the presence of the Philadelphia chromosome, resulting from the reciprocal translocation t(9;22)(q34;q11), which generates the BCR-ABL1 fusion gene encoding a constitutively active tyrosine kinase responsible for uncontrolled proliferation of myeloid cells [2]. In contrast, CLL is a mature B-cell neoplasm characterized by the accumulation of clonal CD5-positive B lymphocytes in peripheral blood, bone marrow, and lymphoid tissues [1,3].

Despite their distinct pathogenesis, several cases describing the coexistence of CLL and CML have been reported in the literature, although the overall number remains very limited [4-6]. In most reported cases, the two malignancies develop sequentially, with CLL preceding the development of CML after several months or years [5]. Less commonly, CML may be diagnosed first, or both diseases may be detected simultaneously during the initial clinical evaluation.

The biological mechanism underlying this coexistence remains unclear. One possible explanation is that the two leukemias arise independently from separate malignant clones, representing a coincidental occurrence in the same patient [6]. Molecular studies have supported this hypothesis by demonstrating that the lymphoid clone typically lacks the BCR-ABL1 fusion gene present in the myeloid lineage [4]. Alternatively, it has been suggested that both malignancies may originate from a common pluripotent hematopoietic stem cell that subsequently undergoes distinct genetic events leading to separate clonal expansions [6].

Another proposed mechanism involves therapy-related clonal evolution, particularly in patients exposed to cytotoxic chemotherapy or targeted therapies. However, the occurrence of both diseases in untreated patients suggests that therapy alone cannot fully explain this association [5].

From a diagnostic perspective, the coexistence of CLL and CML may present significant challenges, particularly when marked leukocytosis with mixed lymphoid and myeloid populations is observed in the peripheral blood. In such cases, comprehensive evaluation, including peripheral blood smear examination, bone marrow analysis, immunophenotyping by flow cytometry, and cytogenetic or molecular testing, is essential for establishing the presence of two distinct clonal disorders [4].

Management strategies in patients with concurrent CLL and CML generally depend on the clinically dominant disease. In many cases, treatment is directed primarily toward CML using tyrosine kinase inhibitors (TKIs) targeting the BCR-ABL1 kinase, while CLL may be managed with observation unless treatment indications arise according to established guidelines [2,3].

Because of the rarity of this dual hematologic malignancy, additional case reports remain valuable for improving understanding of the biological relationship between these disorders and guiding optimal clinical management. The present case adds to the limited literature documenting the coexistence of these two leukemias and highlights the importance of thorough diagnostic evaluation when atypical hematologic findings are encountered.

## Conclusions

Our case illustrates the rare phenomenon of concurrent CLL and CML presenting with life-threatening cytopenia in a patient with multiple comorbidities. This unique presentation underscores the complexity of dual hematological malignancies and emphasizes the need for individualized management strategies. As our understanding of the molecular pathogenesis of these rare entities improves, more targeted therapeutic approaches may emerge to optimize outcomes for these challenging cases.
